# Poly[aqua­(μ_2_-4,4′-bipyridine-κ^2^ *N*:*N*′)(ethane-1,2-diol-κ*O*)(μ_2_-sulfato-κ^2^ *O*:*O*′)nickel(II)]

**DOI:** 10.1107/S1600536813003772

**Published:** 2013-02-16

**Authors:** Kai-Long Zhong

**Affiliations:** a Department of Applied Chemistry, Nanjing College of Chemical Technology, Nanjing 210048, People’s Republic of China

## Abstract

The title compound, [Ni(SO_4_)(C_10_H_8_N_2_)(C_2_H_6_O_2_)(H_2_O)]_*n*_, contains two crystallographically unique Ni^II^ atoms, each lying on a twofold rotation axis and having a slightly distorted octa­hedral environment. It is isotypic with the previously reported Cu^II^ analog [Zhong *et al.* (2011 ▶). *Acta Cryst.* C**67**, m62–m64]. One Ni^II^ atom is coordinated by two N atoms from two bridging 4,4′-bipyridine (4,4′-bipy) ligands, two O atoms from two sulfate ions and two aqua O atoms. The second Ni^II^ atom is surrounded by two N atoms from 4,4′-bipy ligands and four O atoms, two from bridging sulfate ions and from two ethane-1,2-diol ligands. The sulfate anion acts as a bridging ligand, linking adjacent Ni^II^ atoms, leading to the formation of linear ⋯Ni1—Ni2—Ni1—Ni2⋯ chains along the *a*-axis direction. Adjacent chains are further bridged by 4,4′-bipy ligands, resulting in a two-dimensional layered polymer parallel to (001). In the crystal, the polymeric layers are linked by extensive O—H⋯O hydrogen-bonding inter­actions involving the O atoms of the water mol­ecules and the ethane-1,2-diol mol­ecules, resulting in a three-dimensional supra­molecular network.

## Related literature

For Ni-(4,4′-bipy) complexes with perchlorate, citraconate or phthalate anions and a water mol­ecule as a second ligand, see: Yang *et al.* (2003 ▶); Kopf *et al.* (2005 ▶); Wang *et al.* (2006 ▶). For an isotypic structure, see: Zhong *et al.* (2011 ▶). For background to coordination polymers, see: Dietzel *et al.* (2005 ▶); Robin & Fromm (2006 ▶); Sarma *et al.* (2009 ▶); Zhang *et al.* (2010 ▶).
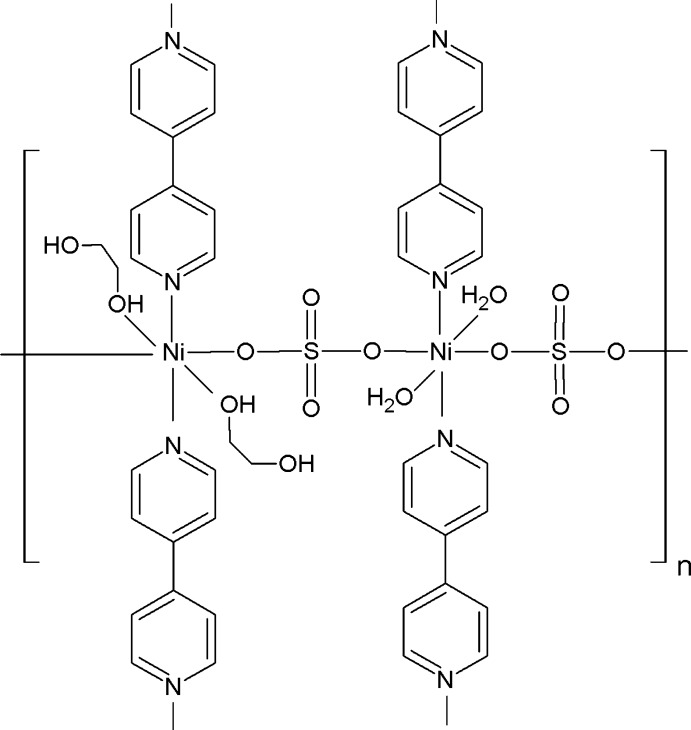


## Experimental

### 

#### Crystal data

[Ni(SO_4_)(C_10_H_8_N_2_)(C_2_H_6_O_2_)(H_2_O)]
*M* *_r_* = 391.04
Monoclinic, 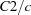

*a* = 11.022 (2) Å
*b* = 22.606 (5) Å
*c* = 12.123 (2) Å
β = 95.65 (3)°
*V* = 3005.9 (10) Å^3^
*Z* = 8
Mo *K*α radiation
μ = 1.47 mm^−1^
*T* = 223 K
0.40 × 0.35 × 0.10 mm

#### Data collection

Rigaku Mercury CCD diffractometer
Absorption correction: multi-scan (*REQAB*; Jacobson, 1998 ▶) *T*_min_ = 0.743, *T*_max_ = 1.000
8555 measured reflections
3420 independent reflections
2885 reflections with *I* > 2σ(*I*)
*R*_int_ = 0.024

#### Refinement

*R*[*F* ^2^ > 2σ(*F* ^2^)] = 0.033
*wR*(*F* ^2^) = 0.086
*S* = 1.06
3420 reflections
214 parameters
H-atom parameters constrained
Δρ_max_ = 0.56 e Å^−3^
Δρ_min_ = −0.41 e Å^−3^

### 

Data collection: *CrystalClear* (Rigaku, 2007 ▶); cell refinement: *CrystalClear*; data reduction: *CrystalClear*; program(s) used to solve structure: *SHELXS97* (Sheldrick, 2008 ▶); program(s) used to refine structure: *SHELXL97* (Sheldrick, 2008 ▶); molecular graphics: *XP* in *SHELXTL* (Sheldrick, 2008 ▶); software used to prepare material for publication: *SHELXTL*.

## Supplementary Material

Crystal structure: contains datablock(s) global, I. DOI: 10.1107/S1600536813003772/zq2195sup1.cif

Structure factors: contains datablock(s) I. DOI: 10.1107/S1600536813003772/zq2195Isup2.hkl

Additional supplementary materials:  crystallographic information; 3D view; checkCIF report

## Figures and Tables

**Table 1:** Selected bond lengths (Å)

Ni1—N2	2.072 (2)
Ni1—O1*W*	2.0809 (15)
Ni1—O1	2.0844 (14)
Ni1—N1	2.101 (2)
Ni2—O5	2.0591 (15)
Ni2—O2	2.0817 (15)
Ni2—N4	2.096 (2)
Ni2—N3	2.101 (2)

**Table 2:** Hydrogen-bond geometry (Å, °)

*D*—H⋯*A*	*D*—H	H⋯*A*	*D*⋯*A*	*D*—H⋯*A*
O6—H6*A*⋯O4^i^	0.82	1.89	2.694 (2)	165
O5—H5*B*⋯O1	0.82	1.82	2.599 (2)	158
O1*W*—H1*WA*⋯O6	0.85	1.86	2.693 (2)	167
O1*W*—H1*WB*⋯O3^ii^	0.85	1.91	2.718 (2)	157

Symmetry codes: (i) 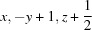; (ii) 
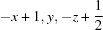
.

**Fig. 1.**
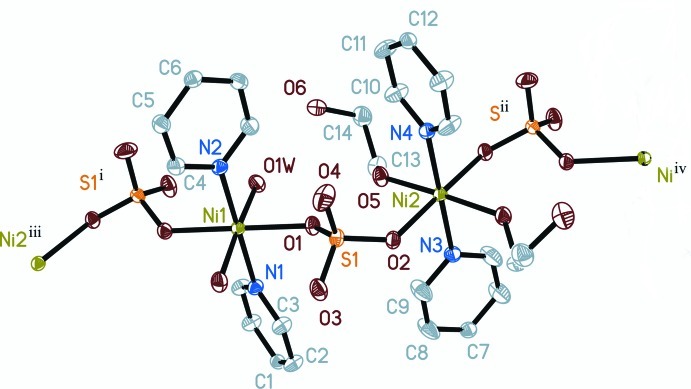
Part of the structure of the title compound, showing the atom-numbering scheme and with displacement ellipsoids drawn at the 35% probability level. All H atoms have been omitted for clarity. Symmetry codes: (i) -x, y, -z + 3/2; (ii) -x + 1, y, -z + 3/2; (iii) x - 1, y, z; (iv) x + 1, y, z.

**Fig. 2.**
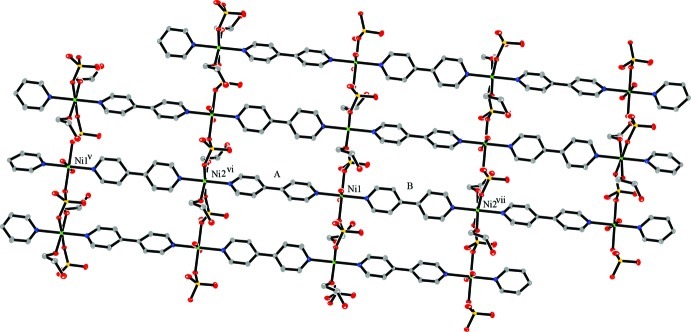
The crystal structure of the title compound viewed along the c axis. All H atoms have omitted for clarity. Symmetry codes: (v) -x + 1, y - 1, z; (vi) x - 1/2, y - 1/2, z; (viii) x - 1/2, y + 1/2, z.

## supplementary crystallographic information

### Comment

Recently, the design and synthesis of metal-organic complexes or polymeric coordination networks belong to a rapidly developing field in coordination and supramolecular chemistry (Dietzel *et al.*, 2005; Robin & Fromm, 2006; Sarma *et al.*, 2009; Zhang *et al.*, 2010). 4,4'-Bipyridine (4,4'-bipy) has been widely used as a bridging ligand to construct interesting complexes. Several Ni-(4,4'-bipy) complexes with perchlorate-anion, citraconate-anion, phthalate-anion and water-molecular ligands have been synthesized and characterized by X-ray diffraction (Yang *et al.*, 2003; Kopf *et al.*, 2005; Wang *et al.*, 2006). The title nickel complex, [Ni_2_(SO_4_)_2_(C_10_H_8_N_2_)_2_(C_2_H_6_O_2_)_2_(H_2_O)_2_]_n_, was obtained *via* a solvothermal reaction.

The single-crystal X-ray diffraction experiment revealed that the title compound is isostructural to the previously reported Cu^II^ analog (Zhong *et al.*, 2011). It contains two crystallographically independent Ni^II^ centres. Atom Ni1 adopts a slightly distorted octahedral geometry. It is coordinated by two N atoms (N1 and N2) from two bridging 4,4'-bipy ligands occupying the axial positions, two O atoms (O1) from two bridging sulfate anions and two O atoms (O1W) from two water molecules occupying the equatorial sites (Fig. 1 & Table 1). The coordination environment of the Ni2 centre is very similar to that of Ni1, with ethane-1,2-diol ligands in place of the water ligands. Both Ni atoms and 4,4'-bipy ligands occupy special positions on crystallographic twofold axes. The Ni—N bond distances [2.072 (2)- 2.101 (2) Å], the Ni—O bond distances [2.0591 (15) - 2.0844 (14) Å] and the *cis* bond angles around Ni^II^ centres [87.20 (4) - 92.80 (4) °] are in agreement with those observed in the previously reported Ni-(4,4'-bipy) complex (Yang *et al.*, 2003). The sulfate anion and 4,4'-bipy act as bridging ligands between two different Ni^2+^ ions, giving rise to the formation of linear ···Ni1—O—SO_2_—O—Ni2—O— SO_2_—O··· chains running along the *a* direction and ···Ni1-bipy-Ni2-bipy··· chains along the *b* direction, respectively. The ···M—O—SO_2_—O—M··· chains and the ···M—bipy—M··· chains are almost orthogonal, leading to a layered structure (Fig. 2). Intermolecular O1W—H5C···O6 and O5—H6···O1 hydrogen bonds help to further stabilize the layered structure (Table 2). In the crystal structure, extensive O—H···O hydrogen-bonding interactions between the water molecules, sulfate anions and 1,2-ethanediol molecules result in a three-dimensional supramolecular network.

### Experimental

Green block-shaped crystals of the title compound were obtained by a procedure similar to that described previously in Zhong *et al.* (2011) with NiSO_4_.7H_2_O instead of CuSO_4_.5H_2_O.

### Refinement

All non-hydrogen atoms were refined anisotropically. The aromatic H atoms were positioned geometrically and allowed to ride on their parent atoms, with C—H = 0.93 Å and *U*_iso_(H) = 1.2*U*_eq_(C). The H atoms of ethane-1,2-diol were geometrically placed and refined using a riding model [O—H = 0.82 Å and C—H = 0.97 Å; *U*_iso_(H) = 1.5*U*_eq_(O) and *U*_iso_(H) = 1.2*U*_eq_(C)]. The water H atoms were either located in difference Fourier maps or placed in calculated positions so as to form a reasonable hydrogen-bond networks, as far as possible. Initially, their positions were refined with tight restraints on the O—H and H···H distances [0.85 (1) and 1.35 (1) Å, respectively] in order to ensure a reasonable geometry. Then they were constrained to ride on their parent O atom [*U*_iso_(H) = 1.5*U*_eq_(O)].

### Crystal data

**Table:**

[Ni(SO_4_)(C_10_H_8_N_2_)(C_2_H_6_O_2_)(H_2_O)]	*F*(000) = 1616
*M**_r_* = 391.04	*D*_x_ = 1.728 Mg m^−^^3^
Monoclinic, *C*2/*c*	Mo *K*α radiation, λ = 0.71073 Å
Hall symbol: -C 2yc	Cell parameters from 6950 reflections
*a* = 11.022 (2) Å	θ = 3.3–27.5°
*b* = 22.606 (5) Å	µ = 1.47 mm^−^^1^
*c* = 12.123 (2) Å	*T* = 223 K
β = 95.65 (3)°	Block, green
*V* = 3005.9 (10) Å^3^	0.40 × 0.35 × 0.10 mm
*Z* = 8	

### Data collection

**Table:**

Rigaku Mercury CCD diffractometer	3420 independent reflections
Radiation source: fine-focus sealed tube	2885 reflections with *I* > 2σ(*I*)
Graphite Monochromator monochromator	*R*_int_ = 0.024
Detector resolution: 28.5714 pixels mm^-1^	θ_max_ = 27.5°, θ_min_ = 3.3°
ω scans	*h* = −14→11
Absorption correction: multi-scan (REQAB; Jacobson, 1998)	*k* = −23→29
*T*_min_ = 0.743, *T*_max_ = 1.000	*l* = −14→15
8555 measured reflections	

### Refinement

**Table:**

Refinement on *F*^2^	Secondary atom site location: difference Fourier map
Least-squares matrix: full	Hydrogen site location: inferred from neighbouring sites
*R*[*F*^2^ > 2σ(*F*^2^)] = 0.033	H-atom parameters constrained
*wR*(*F*^2^) = 0.086	*w* = 1/[σ^2^(*F*_o_^2^) + (0.0501*P*)^2^] where *P* = (*F*_o_^2^ + 2*F*_c_^2^)/3
*S* = 1.06	(Δ/σ)_max_ < 0.001
3420 reflections	Δρ_max_ = 0.56 e Å^−^^3^
214 parameters	Δρ_min_ = −0.41 e Å^−^^3^
0 restraints	Extinction correction: *SHELXL97* (Sheldrick, 2008), Fc^*^=kFc[1+0.001xFc^2^λ^3^/sin(2θ)]^-1/4^
Primary atom site location: structure-invariant direct methods	Extinction coefficient: 0.0014 (2)

### Special details

**Table:**

Geometry. All e.s.d.'s (except the e.s.d. in the dihedral angle between two l.s. planes) are estimated using the full covariance matrix. The cell e.s.d.'s are taken into account individually in the estimation of e.s.d.'s in distances, angles and torsion angles; correlations between e.s.d.'s in cell parameters are only used when they are defined by crystal symmetry. An approximate (isotropic) treatment of cell e.s.d.'s is used for estimating e.s.d.'s involving l.s. planes.
Refinement. Refinement of *F*^2^ against ALL reflections. The weighted *R*-factor *wR* and goodness of fit *S* are based on *F*^2^, conventional *R*-factors *R* are based on *F*, with *F* set to zero for negative *F*^2^. The threshold expression of *F*^2^ > σ(*F*^2^) is used only for calculating *R*-factors(gt) *etc*. and is not relevant to the choice of reflections for refinement. *R*-factors based on *F*^2^ are statistically about twice as large as those based on *F*, and *R*- factors based on ALL data will be even larger.

### Fractional atomic coordinates and isotropic or equivalent isotropic displacement parameters (Å^2^)

**Table:**

	*x*	*y*	*z*	*U*_iso_*/*U*_eq_	
Ni1	0.5000	0.379715 (14)	0.2500	0.01501 (11)	
Ni2	0.0000	0.378572 (13)	0.2500	0.01544 (11)	
S1	0.23450 (4)	0.39830 (2)	0.10920 (4)	0.01777 (13)	
O1	0.31124 (12)	0.37951 (6)	0.21319 (12)	0.0197 (3)	
O1W	0.47576 (13)	0.37522 (5)	0.41787 (12)	0.0234 (3)	
H1WA	0.4323	0.3957	0.4577	0.035*	
H1WB	0.5451	0.3772	0.4551	0.035*	
O2	0.10883 (12)	0.37748 (5)	0.11907 (11)	0.0194 (3)	
O3	0.28195 (13)	0.36860 (7)	0.01488 (13)	0.0289 (4)	
O4	0.23754 (14)	0.46204 (6)	0.09809 (14)	0.0360 (4)	
O5	0.15466 (13)	0.37609 (6)	0.36000 (12)	0.0233 (3)	
H5B	0.2169	0.3765	0.3282	0.028*	
O6	0.30832 (13)	0.43173 (6)	0.52663 (13)	0.0329 (4)	
H6A	0.2989	0.4663	0.5447	0.049*	
N1	0.5000	0.28676 (10)	0.2500	0.0188 (5)	
N2	0.5000	0.47137 (10)	0.2500	0.0182 (5)	
N3	0.0000	0.28565 (10)	0.2500	0.0213 (5)	
N4	0.0000	0.47128 (10)	0.2500	0.0185 (5)	
C1	0.5000	0.16183 (12)	0.2500	0.0188 (6)	
C2	0.43554 (19)	0.19444 (9)	0.16563 (17)	0.0243 (4)	
H2A	0.3912	0.1750	0.1072	0.029*	
C3	0.43763 (19)	0.25555 (9)	0.16891 (18)	0.0245 (4)	
H3A	0.3935	0.2762	0.1119	0.029*	
C4	0.58066 (18)	0.50219 (9)	0.19796 (17)	0.0237 (4)	
H4A	0.6383	0.4815	0.1622	0.028*	
C5	0.58292 (18)	0.56309 (8)	0.19449 (17)	0.0233 (4)	
H5A	0.6395	0.5825	0.1553	0.028*	
C6	0.5000	0.59546 (12)	0.2500	0.0200 (6)	
C7	0.0000	0.16101 (12)	0.2500	0.0215 (6)	
C8	0.0906 (2)	0.19347 (9)	0.2077 (2)	0.0426 (7)	
H8A	0.1540	0.1741	0.1774	0.051*	
C9	0.0880 (2)	0.25446 (10)	0.2101 (2)	0.0419 (6)	
H9A	0.1515	0.2749	0.1821	0.050*	
C10	0.0662 (2)	0.50282 (9)	0.32806 (18)	0.0294 (5)	
H10A	0.1126	0.4824	0.3839	0.035*	
C11	0.0692 (2)	0.56365 (9)	0.33016 (18)	0.0282 (5)	
H11A	0.1180	0.5831	0.3857	0.034*	
C12	0.0000	0.59610 (12)	0.2500	0.0187 (5)	
C13	0.18041 (19)	0.35033 (9)	0.46738 (18)	0.0274 (5)	
H13A	0.2502	0.3242	0.4675	0.033*	
H13B	0.1111	0.3271	0.4854	0.033*	
C14	0.2067 (2)	0.39774 (10)	0.55251 (19)	0.0317 (5)	
H14A	0.1360	0.4232	0.5542	0.038*	
H14B	0.2239	0.3800	0.6252	0.038*	

### Atomic displacement parameters (Å^2^)

**Table:**

	*U*^11^	*U*^22^	*U*^33^	*U*^12^	*U*^13^	*U*^23^
Ni1	0.01408 (18)	0.01323 (17)	0.0177 (2)	0.000	0.00165 (14)	0.000
Ni2	0.01432 (18)	0.01335 (18)	0.0185 (2)	0.000	0.00093 (14)	0.000
S1	0.0155 (2)	0.0190 (2)	0.0187 (2)	−0.00140 (18)	0.00098 (17)	0.00331 (18)
O1	0.0152 (7)	0.0260 (7)	0.0178 (7)	0.0001 (5)	0.0010 (6)	0.0034 (5)
O1W	0.0197 (7)	0.0294 (8)	0.0213 (8)	0.0027 (6)	0.0036 (6)	−0.0021 (6)
O2	0.0146 (6)	0.0232 (7)	0.0202 (7)	−0.0016 (5)	0.0009 (6)	−0.0005 (5)
O3	0.0194 (7)	0.0480 (10)	0.0199 (8)	0.0001 (6)	0.0041 (6)	−0.0026 (6)
O4	0.0342 (8)	0.0204 (7)	0.0514 (11)	−0.0054 (6)	−0.0058 (8)	0.0132 (7)
O5	0.0181 (7)	0.0333 (8)	0.0185 (7)	0.0006 (6)	0.0023 (6)	0.0034 (6)
O6	0.0302 (8)	0.0303 (8)	0.0389 (9)	−0.0006 (6)	0.0072 (7)	−0.0136 (7)
N1	0.0188 (11)	0.0154 (11)	0.0217 (12)	0.000	−0.0005 (9)	0.000
N2	0.0180 (11)	0.0151 (10)	0.0213 (12)	0.000	0.0012 (9)	0.000
N3	0.0220 (11)	0.0133 (10)	0.0288 (13)	0.000	0.0041 (10)	0.000
N4	0.0178 (11)	0.0170 (11)	0.0206 (12)	0.000	0.0021 (9)	0.000
C1	0.0193 (13)	0.0183 (13)	0.0193 (14)	0.000	0.0043 (11)	0.000
C2	0.0290 (10)	0.0186 (9)	0.0235 (11)	−0.0018 (8)	−0.0060 (9)	−0.0013 (8)
C3	0.0284 (10)	0.0197 (10)	0.0240 (11)	−0.0003 (9)	−0.0038 (9)	0.0012 (8)
C4	0.0241 (10)	0.0215 (10)	0.0259 (11)	−0.0009 (8)	0.0050 (9)	−0.0019 (8)
C5	0.0241 (10)	0.0202 (9)	0.0268 (11)	−0.0009 (8)	0.0076 (9)	0.0021 (8)
C6	0.0220 (13)	0.0174 (13)	0.0201 (14)	0.000	−0.0003 (11)	0.000
C7	0.0230 (14)	0.0190 (13)	0.0226 (15)	0.000	0.0021 (11)	0.000
C8	0.0407 (13)	0.0191 (10)	0.0735 (19)	0.0030 (10)	0.0328 (14)	−0.0012 (12)
C9	0.0388 (13)	0.0214 (10)	0.0711 (19)	−0.0001 (10)	0.0327 (13)	0.0020 (12)
C10	0.0376 (12)	0.0189 (10)	0.0287 (12)	0.0023 (9)	−0.0120 (10)	−0.0003 (9)
C11	0.0351 (12)	0.0214 (10)	0.0254 (11)	−0.0019 (9)	−0.0106 (9)	−0.0030 (8)
C12	0.0191 (13)	0.0158 (12)	0.0217 (14)	0.000	0.0041 (11)	0.000
C13	0.0284 (11)	0.0268 (11)	0.0265 (11)	−0.0012 (9)	0.0003 (9)	0.0094 (9)
C14	0.0303 (11)	0.0416 (13)	0.0240 (11)	−0.0020 (10)	0.0066 (9)	−0.0008 (10)

### Geometric parameters (Å, º)

**Table:**

Ni1—N2	2.072 (2)	C1—C2^i^	1.397 (2)
Ni1—O1W	2.0809 (15)	C1—C2	1.397 (2)
Ni1—O1W^i^	2.0809 (15)	C1—C12^iii^	1.486 (4)
Ni1—O1	2.0844 (14)	C2—C3	1.382 (3)
Ni1—O1^i^	2.0844 (14)	C2—H2A	0.9300
Ni1—N1	2.101 (2)	C3—H3A	0.9300
Ni2—O5	2.0591 (15)	C4—C5	1.378 (3)
Ni2—O5^ii^	2.0591 (15)	C4—H4A	0.9300
Ni2—O2^ii^	2.0817 (15)	C5—C6	1.395 (2)
Ni2—O2	2.0817 (15)	C5—H5A	0.9300
Ni2—N4	2.096 (2)	C6—C5^i^	1.395 (2)
Ni2—N3	2.101 (2)	C6—C7^iv^	1.482 (4)
S1—O4	1.4480 (15)	C7—C8	1.378 (3)
S1—O3	1.4660 (16)	C7—C8^ii^	1.378 (3)
S1—O2	1.4788 (14)	C7—C6^v^	1.482 (4)
S1—O1	1.5084 (15)	C8—C9	1.379 (3)
O1W—H1WA	0.8500	C8—H8A	0.9300
O1W—H1WB	0.8500	C9—H9A	0.9300
O5—C13	1.429 (2)	C10—C11	1.376 (3)
O5—H5B	0.8200	C10—H10A	0.9300
O6—C14	1.419 (3)	C11—C12	1.385 (2)
O6—H6A	0.8200	C11—H11A	0.9300
N1—C3	1.343 (2)	C12—C11^ii^	1.385 (2)
N1—C3^i^	1.343 (2)	C12—C1^vi^	1.486 (4)
N2—C4^i^	1.336 (2)	C13—C14	1.496 (3)
N2—C4	1.336 (2)	C13—H13A	0.9700
N3—C9^ii^	1.328 (3)	C13—H13B	0.9700
N3—C9	1.328 (3)	C14—H14A	0.9700
N4—C10	1.342 (2)	C14—H14B	0.9700
N4—C10^ii^	1.342 (2)		
			
N2—Ni1—O1W	92.80 (4)	C9—N3—Ni2	122.06 (13)
N2—Ni1—O1W^i^	92.80 (4)	C10—N4—C10^ii^	115.8 (2)
O1W—Ni1—O1W^i^	174.41 (7)	C10—N4—Ni2	122.10 (12)
N2—Ni1—O1	90.13 (4)	C10^ii^—N4—Ni2	122.10 (12)
O1W—Ni1—O1	89.30 (6)	C2^i^—C1—C2	116.3 (2)
O1W^i^—Ni1—O1	90.69 (6)	C2^i^—C1—C12^iii^	121.85 (12)
N2—Ni1—O1^i^	90.13 (4)	C2—C1—C12^iii^	121.85 (12)
O1W—Ni1—O1^i^	90.69 (6)	C3—C2—C1	120.03 (18)
O1W^i^—Ni1—O1^i^	89.30 (6)	C3—C2—H2A	120.0
O1—Ni1—O1^i^	179.74 (7)	C1—C2—H2A	120.0
N2—Ni1—N1	180.0	N1—C3—C2	123.50 (18)
O1W—Ni1—N1	87.20 (4)	N1—C3—H3A	118.2
O1W^i^—Ni1—N1	87.20 (4)	C2—C3—H3A	118.2
O1—Ni1—N1	89.87 (4)	N2—C4—C5	123.4 (2)
O1^i^—Ni1—N1	89.87 (4)	N2—C4—H4A	118.3
O5—Ni2—O5^ii^	176.88 (7)	C5—C4—H4A	118.3
O5—Ni2—O2^ii^	90.48 (6)	C4—C5—C6	119.7 (2)
O5^ii^—Ni2—O2^ii^	89.49 (6)	C4—C5—H5A	120.2
O5—Ni2—O2	89.49 (6)	C6—C5—H5A	120.2
O5^ii^—Ni2—O2	90.48 (6)	C5^i^—C6—C5	116.7 (3)
O2^ii^—Ni2—O2	178.64 (7)	C5^i^—C6—C7^iv^	121.66 (13)
O5—Ni2—N4	91.56 (4)	C5—C6—C7^iv^	121.66 (13)
O5^ii^—Ni2—N4	91.56 (4)	C8—C7—C8^ii^	115.6 (3)
O2^ii^—Ni2—N4	90.68 (3)	C8—C7—C6^v^	122.18 (13)
O2—Ni2—N4	90.68 (3)	C8^ii^—C7—C6^v^	122.18 (13)
O5—Ni2—N3	88.44 (4)	C7—C8—C9	120.5 (2)
O5^ii^—Ni2—N3	88.44 (4)	C7—C8—H8A	119.8
O2^ii^—Ni2—N3	89.32 (3)	C9—C8—H8A	119.8
O2—Ni2—N3	89.32 (3)	N3—C9—C8	123.7 (2)
N4—Ni2—N3	180.0	N3—C9—H9A	118.1
O4—S1—O3	111.71 (10)	C8—C9—H9A	118.1
O4—S1—O2	110.77 (8)	N4—C10—C11	123.70 (19)
O3—S1—O2	109.04 (9)	N4—C10—H10A	118.2
O4—S1—O1	109.99 (8)	C11—C10—H10A	118.2
O3—S1—O1	108.01 (8)	C10—C11—C12	120.37 (19)
O2—S1—O1	107.18 (8)	C10—C11—H11A	119.8
S1—O1—Ni1	130.10 (9)	C12—C11—H11A	119.8
Ni1—O1W—H1WA	131.5	C11^ii^—C12—C11	116.1 (3)
Ni1—O1W—H1WB	108.7	C11^ii^—C12—C1^vi^	121.97 (13)
H1WA—O1W—H1WB	101.4	C11—C12—C1^vi^	121.97 (13)
S1—O2—Ni2	132.26 (9)	O5—C13—C14	110.12 (17)
C13—O5—Ni2	132.74 (12)	O5—C13—H13A	109.6
C13—O5—H5B	109.5	C14—C13—H13A	109.6
Ni2—O5—H5B	111.9	O5—C13—H13B	109.6
C14—O6—H6A	109.5	C14—C13—H13B	109.6
C3—N1—C3^i^	116.6 (2)	H13A—C13—H13B	108.2
C3—N1—Ni1	121.69 (12)	O6—C14—C13	109.80 (18)
C3^i^—N1—Ni1	121.69 (12)	O6—C14—H14A	109.7
C4^i^—N2—C4	117.1 (2)	C13—C14—H14A	109.7
C4^i^—N2—Ni1	121.44 (12)	O6—C14—H14B	109.7
C4—N2—Ni1	121.44 (12)	C13—C14—H14B	109.7
C9^ii^—N3—C9	115.9 (3)	H14A—C14—H14B	108.2
C9^ii^—N3—Ni2	122.06 (13)		
			
O4—S1—O1—Ni1	−70.98 (12)	O2^ii^—Ni2—N3—C9^ii^	24.04 (16)
O3—S1—O1—Ni1	51.16 (12)	O2—Ni2—N3—C9^ii^	−155.96 (16)
O2—S1—O1—Ni1	168.52 (9)	O5—Ni2—N3—C9	−65.47 (16)
N2—Ni1—O1—S1	68.28 (10)	O5^ii^—Ni2—N3—C9	114.53 (16)
O1W—Ni1—O1—S1	161.08 (10)	O2^ii^—Ni2—N3—C9	−155.96 (16)
O1W^i^—Ni1—O1—S1	−24.52 (10)	O2—Ni2—N3—C9	24.04 (16)
N1—Ni1—O1—S1	−111.72 (10)	O5—Ni2—N4—C10	−15.81 (13)
O4—S1—O2—Ni2	−76.72 (13)	O5^ii^—Ni2—N4—C10	164.19 (13)
O3—S1—O2—Ni2	159.96 (10)	O2^ii^—Ni2—N4—C10	74.68 (13)
O1—S1—O2—Ni2	43.28 (12)	O2—Ni2—N4—C10	−105.32 (13)
O5—Ni2—O2—S1	−27.71 (11)	O5—Ni2—N4—C10^ii^	164.19 (13)
O5^ii^—Ni2—O2—S1	155.42 (10)	O5^ii^—Ni2—N4—C10^ii^	−15.81 (13)
N4—Ni2—O2—S1	63.85 (10)	O2^ii^—Ni2—N4—C10^ii^	−105.32 (13)
N3—Ni2—O2—S1	−116.15 (10)	O2—Ni2—N4—C10^ii^	74.68 (13)
O2^ii^—Ni2—O5—C13	31.31 (16)	C2^i^—C1—C2—C3	−0.18 (15)
O2—Ni2—O5—C13	−147.33 (16)	C12^iii^—C1—C2—C3	179.82 (15)
N4—Ni2—O5—C13	122.01 (16)	C3^i^—N1—C3—C2	−0.20 (16)
N3—Ni2—O5—C13	−57.99 (16)	Ni1—N1—C3—C2	179.80 (16)
O1W—Ni1—N1—C3	135.88 (12)	C1—C2—C3—N1	0.4 (3)
O1W^i^—Ni1—N1—C3	−44.12 (12)	C4^i^—N2—C4—C5	−0.94 (14)
O1—Ni1—N1—C3	46.58 (12)	Ni1—N2—C4—C5	179.06 (14)
O1^i^—Ni1—N1—C3	−133.42 (12)	N2—C4—C5—C6	1.9 (3)
O1W—Ni1—N1—C3^i^	−44.12 (12)	C4—C5—C6—C5^i^	−0.87 (13)
O1W^i^—Ni1—N1—C3^i^	135.88 (12)	C4—C5—C6—C7^iv^	179.13 (13)
O1—Ni1—N1—C3^i^	−133.42 (12)	C8^ii^—C7—C8—C9	0.6 (2)
O1^i^—Ni1—N1—C3^i^	46.58 (12)	C6^v^—C7—C8—C9	−179.4 (2)
O1W—Ni1—N2—C4^i^	−43.57 (11)	C9^ii^—N3—C9—C8	0.6 (2)
O1W^i^—Ni1—N2—C4^i^	136.43 (11)	Ni2—N3—C9—C8	−179.4 (2)
O1—Ni1—N2—C4^i^	45.73 (11)	C7—C8—C9—N3	−1.2 (4)
O1^i^—Ni1—N2—C4^i^	−134.27 (11)	C10^ii^—N4—C10—C11	−0.58 (17)
O1W—Ni1—N2—C4	136.43 (11)	Ni2—N4—C10—C11	179.42 (17)
O1W^i^—Ni1—N2—C4	−43.57 (11)	N4—C10—C11—C12	1.2 (3)
O1—Ni1—N2—C4	−134.27 (11)	C10—C11—C12—C11^ii^	−0.54 (16)
O1^i^—Ni1—N2—C4	45.73 (11)	C10—C11—C12—C1^vi^	179.46 (16)
O5—Ni2—N3—C9^ii^	114.53 (16)	Ni2—O5—C13—C14	−114.44 (17)
O5^ii^—Ni2—N3—C9^ii^	−65.47 (16)	O5—C13—C14—O6	−59.5 (2)

Symmetry codes: (i) −*x*+1, *y*, −*z*+1/2; (ii) −*x*, *y*, −*z*+1/2; (iii) *x*+1/2, *y*−1/2, *z*; (iv) *x*+1/2, *y*+1/2, *z*; (v) *x*−1/2, *y*−1/2, *z*; (vi) *x*−1/2, *y*+1/2, *z*.

### Hydrogen-bond geometry (Å, º)

**Table:**

*D*—H···*A*	*D*—H	H···*A*	*D*···*A*	*D*—H···*A*
O6—H6*A*···O4^vii^	0.82	1.89	2.694 (2)	165
O5—H5*B*···O1	0.82	1.82	2.599 (2)	158
O1*W*—H1*WA*···O6	0.85	1.86	2.693 (2)	167
O1*W*—H1*WB*···O3^i^	0.85	1.91	2.718 (2)	157

Symmetry codes: (i) −*x*+1, *y*, −*z*+1/2; (vii) *x*, −*y*+1, *z*+1/2.
